# 3-(4-Biphenyl-1-yl)-3-hydr­oxy-1-phenyl­prop-2-en-1-one

**DOI:** 10.1107/S1600536808042566

**Published:** 2008-12-20

**Authors:** Chunyang Zheng, Dunjia Wang, Ling Fan

**Affiliations:** a Hubei Key Laboratory of Bioanalytical Techniques, Hubei Normal University, Huangshi 435002, People’s Republic of China; b College of Chemistry and Environmental Engineering, Hubei Normal University, Huangshi 435002, People’s Republic of China

## Abstract

In the title compound, C_21_H_16_O_2_, the six crystallographically independent mol­ecules (*Z*′ = 6) all exist in the enolized form. Strong intra­molecular hydrogen bonds are observed: one approximate H-atom-centered O⋯H⋯O hydrogen bond, two tautomeric forms O—H⋯O (three mol­ecules) and O⋯H—O (two mol­ecules). Only one weak inter­molecular C—H⋯O hydrogen bond between two neighboring mol­ecules is observed in the crystal structure. In addition, eight very weak non-conventional inter­molecular C—H⋯π hydrogen-bonding contacts between mol­ecules are observed.

## Related literature

For proton transfer in solid 1-phenyl­butane-1,3-dione and related 1,3-diones, see: Vila *et al.* (1991 ▶). For the crystal structures of eight intra­molecular hydrogen-bonded 1,3-diaryl-1,3-propane­dione enols, see: Bertolasi *et al.* (1991 ▶). For a discussion of the covalent *versus* the electrostatic nature of the strong hydrogen bond, see: Gilli *et al.* (2004 ▶). For electron transfer reactions of aromatic α,β-ep­oxy ketones, see: Hasegawa *et al.* (1997 ▶). For 1,3-diketones used as ligands, see: Jang *et al.* (2006 ▶). For weak hydrogen bonds, see: Desiraju & Steiner (2001 ▶).
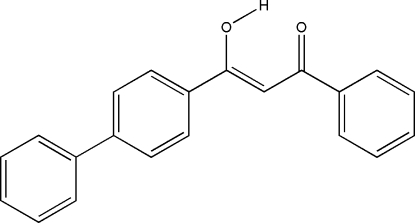


## Experimental

### 

#### Crystal data

C_21_H_16_O_2_
*M* *_r_* = 300.34
Triclinic, 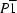

*a* = 10.6087 (13) Å
*b* = 17.814 (2) Å
*c* = 26.394 (3) Å
α = 72.170 (2)°
β = 86.069 (2)°
γ = 89.450 (2)°
*V* = 4736.8 (10) Å^3^
*Z* = 12
Mo *K*α radiation
μ = 0.08 mm^−1^
*T* = 298 (2) K
0.20 × 0.10 × 0.10 mm

#### Data collection

Bruker SMART CCD area-detector diffractometer
Absorption correction: multi-scan (*SADABS*; Sheldrick, 1996 ▶) *T*_min_ = 0.980, *T*_max_ = 0.989
55512 measured reflections
16514 independent reflections
9991 reflections with *I* > 2σ(*I*)
*R*_int_ = 0.034

#### Refinement

*R*[*F* ^2^ > 2σ(*F* ^2^)] = 0.078
*wR*(*F* ^2^) = 0.154
*S* = 1.07
16514 reflections
1255 parameters
H atoms treated by a mixture of independent and constrained refinement
Δρ_max_ = 0.19 e Å^−3^
Δρ_min_ = −0.16 e Å^−3^

### 

Data collection: *SMART* (Bruker, 1997 ▶); cell refinement: *SAINT* (Bruker, 1999 ▶); data reduction: *SAINT*; program(s) used to solve structure: *SHELXS97* (Sheldrick, 2008 ▶); program(s) used to refine structure: *SHELXL97* (Sheldrick, 2008 ▶); molecular graphics: *SHELXTL* (Sheldrick, 2008 ▶); software used to prepare material for publication: *SHELXTL* and *PLATON* (Spek, 2003 ▶).

## Supplementary Material

Crystal structure: contains datablocks global, I. DOI: 10.1107/S1600536808042566/si2145sup1.cif

Structure factors: contains datablocks I. DOI: 10.1107/S1600536808042566/si2145Isup2.hkl

Additional supplementary materials:  crystallographic information; 3D view; checkCIF report

## Figures and Tables

**Table 1:** Hydrogen-bond geometry (Å, °)

*D*—H⋯*A*	*D*—H	H⋯*A*	*D*⋯*A*	*D*—H⋯*A*
O1—H1*A*⋯O2	1.20 (5)	1.33 (5)	2.480 (4)	156 (4)
O3—H3*A*⋯O4	1.21 (4)	1.33 (4)	2.479 (4)	156 (3)
O5—H5*A*⋯O6	1.33 (5)	1.22 (5)	2.474 (4)	152 (4)
O7—H7*A*⋯O8	1.28 (5)	1.26 (5)	2.465 (4)	152 (4)
O9—H9*A*⋯O10	1.20 (5)	1.34 (5)	2.473 (4)	154 (3)
O11—H11*A*⋯O12	1.33 (5)	1.25 (5)	2.494 (4)	152 (4)
C122—H122⋯O10^i^	0.93	2.58	3.429 (5)	152
C19—H19⋯*Cg*10^ii^	0.93	2.93	3.739 (5)	147
C23—H23⋯*Cg*17^iii^	0.93	2.90	3.714 (4)	146
C32—H32⋯*Cg*17^iv^	0.93	2.94	3.749 (4)	147
C39—H39⋯*Cg*3^i^	0.93	2.82	3.674 (5)	152
C48—H48⋯*Cg*11^v^	0.93	2.79	3.618 (4)	149
C69—H69⋯*Cg*8^i^	0.93	2.95	3.820 (4)	155
C93—H93⋯*Cg*2^vi^	0.93	3.00	3.692 (3)	133
C107—H107⋯*Cg*14^i^	0.93	2.83	3.670 (4)	151

Symmetry codes: (i) 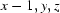; (ii) 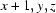; (iii) 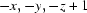; (iv) 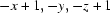; (v) 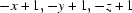; (vi) 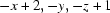. *Cg*2 is the centroid of atoms C7–C12, *Cg*3 of atoms C16–C21, *Cg*8 of atoms C49–C54, *Cg*10 of atoms C64–C69, *Cg*11 of atoms C70–C75, *Cg*14 of atoms C91–C96 and *Cg*17 of atoms C112–C117.

**Fig. 1.**
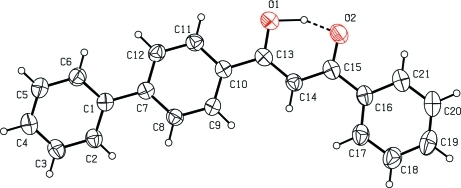
A view of the molecule 1 out of six independent molecules of the title compound, showing the atom-labeling scheme for non-hydrogen atoms. Displacement ellipsoids are drawn at the 50% probability level. It is difficult to show all molecules with atom labels. The dashed line indicates an intramolecular hydrogen bond.

**Fig. 2.**
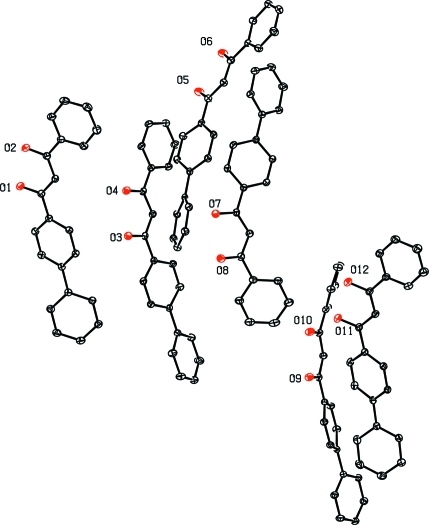
A view of the six independent molecules, only O atoms were labelled and all H atoms have been omitted for clarity.

## supplementary crystallographic information

### Comment

Both in solution and in the solid state, considerable attention has been focused on the structures of 1,3-diketones due to their enolic tautomeric forms and their ability to form strong intermolecular or intramolecular hydrogen bonds (Vila *et al.*, 1991; Bertolasi *et al.*, 1991; Gilli *et al.*, 2004). These compounds posses sometimes unique chemical properties, which make them extremely attractive as intermediates in syntheses (Hasegawa *et al.*, 1997). They are also used widely in the chemistry of metallocomplexes (Jang *et al.*, 2006).

The crystal structure of the title compound (Fig. 1), has six independent molecules which all exist in the enol form, stabilized by intramolecular hydrogen bonds. The O—H distances of enol six ring and dihedral angles in six independent molecules are different. In molecule 4, the intramolecular O···H···O hydrogen bond can be described as approximately proton-centered, and the others as dynamic or static mixtures (Gilli *et al.*, 2004) of two tautomeric O—H···O and O···H—O forms. Molecules 1, 2, 5 belong to O—H···O, and molecules 3 and 6 to the O···H—H tautomer (Table 1). In addition, there is only one weak intermolecular hydrogen bond between molecules 5 and 6, and other pairs of molecules do not show any C—H···O contacts, but probably C—H···π interactions (Table 1). Details of such non-conventional π contacts are reviewed by Desiraju & Steiner (2001). The following Cgs in Table 1 are the centroids of the π acceptor ring systems: Cg2 = C7 - C12, Cg3 = C16 - C21, Cg8 = C49 - C54, Cg10 = C64 - C69, Cg11 = C70 - C75, Cg14 = C91 - C96, Cg17 = C112 - C117. The central benzene ring in molecule 1 (C7 - C12) makes dihedral angles of 28.04 (8) and 9.01 (7) ° with the benzene rings C1 - C6 and C16 - C21, respectively; in the other five molecules, the corresponding angles are 18.49 (3) and 5.74 (2) °, 30.57 (5) and 12.48 (4) °, 26.58 (4) and 29.54 (3) °, 25.09 (5) and 28.07 (3) °, 27.68 (2) and 13.52 (4) °, respectively.

### Experimental

1-(4-bilphenyl)ethanone (7.84 g, 0.04 mol), ethyl benzoate (6.02 g, 0.04 mol), NaNH_2_ (1.95 g, 0.05 mol) and dry ether (100 ml) were placed into round bottom flask. The mixture was stirred 6 h at room temperature under a blanket of nitrogen, acidified with dilute hydrochloric acid, and stirring was continued until all solids dissolved. The ether layer was separated and washed with saturated NaHCO_3_ solution, dried over anhydrous Na_2_SO_4_ and was removed by evaporation. The residual solid was recrystallized from ethanol solution to give the title compound (yield 4.60 g, 38.3%, m.p. 383 K). Crystals suitable for X-ray diffraction were grown by slow evaporation of the CHCl_3_—EtOH (1:4) solutions at room temperature.

### Refinement

H atoms were placed in geometrically idealized positions and constrained to ride on their parent atoms, with C—H distances of 0.93 Å, and with *U*_iso_(H) = 1.2 *U*_eq_(C). The H atoms of the hydroxyl groups were located in a difference Fourier map and their positions were refined freely with *U*_iso_(H) = 1.5 *U*_iso_(O).

### Crystal data

**Table:**

C_21_H_16_O_2_	*Z* = 12
*M**_r_* = 300.34	*F*(000) = 1896
Triclinic, *P*1	*D*_x_ = 1.263 Mg m^−^^3^
Hall symbol: -P 1	Melting point: 383 K
*a* = 10.6087 (13) Å	Mo *K*α radiation, λ = 0.71073 Å
*b* = 17.814 (2) Å	Cell parameters from 3181 reflections
*c* = 26.394 (3) Å	θ = 2.3–21.5°
α = 72.170 (2)°	µ = 0.08 mm^−^^1^
β = 86.069 (2)°	*T* = 298 K
γ = 89.450 (2)°	Block, colorless
*V* = 4736.8 (10) Å^3^	0.20 × 0.10 × 0.10 mm

### Data collection

**Table:**

Bruker SMART CCD area-detector diffractometer	16514 independent reflections
Radiation source: fine-focus sealed tube	9991 reflections with *I* > 2σ(*I*)
graphite	*R*_int_ = 0.034
φ and ω scans	θ_max_ = 25.0°, θ_min_ = 1.6°
Absorption correction: multi-scan (*SADABS*; Sheldrick, 1996)	*h* = −12→12
*T*_min_ = 0.980, *T*_max_ = 0.989	*k* = −21→19
55512 measured reflections	*l* = −30→31

### Refinement

**Table:**

Refinement on *F*^2^	Primary atom site location: structure-invariant direct methods
Least-squares matrix: full	Secondary atom site location: difference Fourier map
*R*[*F*^2^ > 2σ(*F*^2^)] = 0.078	Hydrogen site location: inferred from neighbouring sites
*wR*(*F*^2^) = 0.154	H atoms treated by a mixture of independent and constrained refinement
*S* = 1.07	*w* = 1/[σ^2^(*F*_o_^2^) + (0.0276*P*)^2^ + 3.4135*P*] where *P* = (*F*_o_^2^ + 2*F*_c_^2^)/3
16514 reflections	(Δ/σ)_max_ = 0.001
1255 parameters	Δρ_max_ = 0.19 e Å^−^^3^
0 restraints	Δρ_min_ = −0.16 e Å^−^^3^

### Special details

**Table:**

Geometry. All e.s.d.'s (except the e.s.d. in the dihedral angle between two l.s. planes) are estimated using the full covariance matrix. The cell e.s.d.'s are taken into account individually in the estimation of e.s.d.'s in distances, angles and torsion angles; correlations between e.s.d.'s in cell parameters are only used when they are defined by crystal symmetry. An approximate (isotropic) treatment of cell e.s.d.'s is used for estimating e.s.d.'s involving l.s. planes.
Refinement. Refinement of *F*^2^ against ALL reflections. The weighted *R*-factor *wR* and goodness of fit *S* are based on *F*^2^, conventional *R*-factors *R* are based on *F*, with *F* set to zero for negative *F*^2^. The threshold expression of *F*^2^ > σ(*F*^2^) is used only for calculating *R*-factors(gt) *etc*. and is not relevant to the choice of reflections for refinement. *R*-factors based on *F*^2^ are statistically about twice as large as those based on *F*, and *R*- factors based on ALL data will be even larger.

### Fractional atomic coordinates and isotropic or equivalent isotropic displacement parameters (Å^2^)

**Table:**

	*x*	*y*	*z*	*U*_iso_*/*U*_eq_	
C1	0.5878 (3)	−0.04592 (19)	0.92533 (13)	0.0567 (8)	
C2	0.5157 (3)	−0.0527 (2)	0.88522 (14)	0.0655 (9)	
H2	0.4953	−0.0075	0.8584	0.079*	
C3	0.4738 (3)	−0.1249 (2)	0.88418 (15)	0.0744 (10)	
H3	0.4253	−0.1280	0.8568	0.089*	
C4	0.5028 (4)	−0.1925 (2)	0.92335 (17)	0.0798 (11)	
H4	0.4742	−0.2414	0.9227	0.096*	
C5	0.5745 (4)	−0.1871 (2)	0.96328 (16)	0.0826 (11)	
H5	0.5947	−0.2326	0.9899	0.099*	
C6	0.6170 (3)	−0.1146 (2)	0.96440 (14)	0.0693 (9)	
H6	0.6659	−0.1119	0.9917	0.083*	
C7	0.6346 (3)	0.03222 (18)	0.92581 (13)	0.0553 (8)	
C8	0.6563 (3)	0.09381 (19)	0.87890 (13)	0.0615 (8)	
H8	0.6389	0.0864	0.8467	0.074*	
C9	0.7029 (3)	0.16578 (18)	0.87886 (13)	0.0600 (8)	
H9	0.7172	0.2056	0.8466	0.072*	
C10	0.7289 (3)	0.18012 (19)	0.92559 (13)	0.0574 (8)	
C11	0.7056 (3)	0.1192 (2)	0.97302 (13)	0.0641 (9)	
H11	0.7211	0.1272	1.0052	0.077*	
C12	0.6600 (3)	0.0468 (2)	0.97290 (13)	0.0642 (9)	
H12	0.6459	0.0070	1.0051	0.077*	
C13	0.7833 (3)	0.2558 (2)	0.92644 (15)	0.0622 (9)	
C14	0.7991 (3)	0.32151 (19)	0.88152 (14)	0.0618 (9)	
H14	0.7713	0.3193	0.8494	0.074*	
C15	0.8558 (3)	0.3906 (2)	0.88353 (15)	0.0655 (9)	
C16	0.8740 (3)	0.46205 (19)	0.83683 (15)	0.0644 (9)	
C17	0.8367 (3)	0.4650 (2)	0.78706 (16)	0.0777 (10)	
H17	0.8013	0.4205	0.7820	0.093*	
C18	0.8517 (4)	0.5335 (3)	0.74485 (18)	0.0937 (13)	
H18	0.8252	0.5349	0.7117	0.112*	
C19	0.9054 (4)	0.5995 (2)	0.7512 (2)	0.0959 (13)	
H19	0.9160	0.6454	0.7226	0.115*	
C20	0.9433 (4)	0.5968 (2)	0.8004 (2)	0.0927 (13)	
H20	0.9796	0.6413	0.8051	0.111*	
C21	0.9280 (3)	0.5292 (2)	0.84268 (17)	0.0804 (11)	
H21	0.9542	0.5283	0.8758	0.097*	
C22	0.2752 (3)	−0.08517 (18)	0.73658 (12)	0.0566 (8)	
C23	0.1476 (3)	−0.1027 (2)	0.74626 (16)	0.0811 (11)	
H23	0.0934	−0.0672	0.7558	0.097*	
C24	0.0991 (4)	−0.1718 (2)	0.74203 (19)	0.0989 (14)	
H24	0.0128	−0.1823	0.7488	0.119*	
C25	0.1759 (4)	−0.2250 (2)	0.72810 (16)	0.0860 (12)	
H25	0.1422	−0.2714	0.7251	0.103*	
C26	0.3026 (4)	−0.2099 (2)	0.71852 (15)	0.0828 (11)	
H26	0.3560	−0.2460	0.7092	0.099*	
C27	0.3508 (3)	−0.1405 (2)	0.72289 (14)	0.0750 (10)	
H27	0.4373	−0.1307	0.7164	0.090*	
C28	0.3293 (3)	−0.00972 (18)	0.73938 (11)	0.0530 (8)	
C29	0.2542 (3)	0.0553 (2)	0.73719 (14)	0.0699 (10)	
H29	0.1677	0.0513	0.7345	0.084*	
C30	0.3040 (3)	0.1256 (2)	0.73883 (14)	0.0668 (9)	
H30	0.2510	0.1682	0.7368	0.080*	
C31	0.4318 (3)	0.13387 (18)	0.74341 (12)	0.0533 (8)	
C32	0.5075 (3)	0.06965 (19)	0.74514 (13)	0.0626 (9)	
H32	0.5941	0.0738	0.7475	0.075*	
C33	0.4568 (3)	−0.00013 (19)	0.74349 (13)	0.0628 (9)	
H33	0.5102	−0.0424	0.7452	0.075*	
C34	0.4883 (3)	0.20721 (18)	0.74734 (11)	0.0537 (8)	
C35	0.4209 (3)	0.27266 (19)	0.75000 (12)	0.0598 (8)	
H35	0.3335	0.2721	0.7488	0.072*	
C36	0.4808 (3)	0.34033 (19)	0.75456 (12)	0.0577 (8)	
C37	0.4095 (3)	0.41092 (18)	0.75769 (12)	0.0549 (8)	
C38	0.2800 (3)	0.4143 (2)	0.75820 (15)	0.0763 (10)	
H38	0.2346	0.3710	0.7562	0.092*	
C39	0.2158 (4)	0.4804 (2)	0.76161 (17)	0.0897 (12)	
H39	0.1280	0.4811	0.7625	0.108*	
C40	0.2813 (4)	0.5451 (2)	0.76369 (15)	0.0813 (11)	
H40	0.2384	0.5900	0.7657	0.098*	
C41	0.4104 (4)	0.5433 (2)	0.76286 (14)	0.0765 (10)	
H41	0.4553	0.5872	0.7641	0.092*	
C42	0.47451 (17)	0.47661 (11)	0.76022 (7)	0.0677 (9)	
H42	0.5622	0.4758	0.7601	0.081*	
C43	0.66144 (17)	0.40465 (11)	0.57063 (7)	0.0542 (8)	
C44	0.61617 (17)	0.33663 (11)	0.60936 (7)	0.0605 (8)	
H44	0.5620	0.3412	0.6375	0.073*	
C45	0.6498 (3)	0.2625 (2)	0.60731 (14)	0.0706 (10)	
H45	0.6176	0.2180	0.6337	0.085*	
C46	0.7308 (3)	0.2542 (2)	0.56646 (15)	0.0715 (10)	
H46	0.7542	0.2044	0.5651	0.086*	
C47	0.7767 (3)	0.3206 (2)	0.52771 (14)	0.0708 (9)	
H47	0.8315	0.3156	0.4999	0.085*	
C48	0.7422 (3)	0.3947 (2)	0.52958 (13)	0.0639 (9)	
H48	0.7738	0.4389	0.5027	0.077*	
C49	0.6198 (3)	0.48401 (18)	0.57178 (12)	0.0546 (8)	
C50	0.5856 (3)	0.4987 (2)	0.61940 (13)	0.0668 (9)	
H50	0.5953	0.4593	0.6514	0.080*	
C51	0.5372 (3)	0.5705 (2)	0.62040 (13)	0.0681 (9)	
H51	0.5135	0.5784	0.6530	0.082*	
C52	0.5238 (3)	0.63086 (19)	0.57366 (13)	0.0580 (8)	
C53	0.5617 (3)	0.61754 (19)	0.52591 (13)	0.0630 (9)	
H53	0.5558	0.6578	0.4940	0.076*	
C54	0.6079 (3)	0.54530 (19)	0.52530 (13)	0.0619 (8)	
H54	0.6319	0.5375	0.4927	0.074*	
C55	0.4654 (3)	0.7057 (2)	0.57609 (14)	0.0631 (9)	
C56	0.4660 (3)	0.77418 (19)	0.53215 (13)	0.0631 (9)	
H56	0.5098	0.7746	0.5003	0.076*	
C57	0.4030 (3)	0.8408 (2)	0.53510 (14)	0.0636 (9)	
C58	0.3959 (3)	0.91392 (19)	0.48994 (13)	0.0582 (8)	
C59	0.3344 (3)	0.9786 (2)	0.49812 (15)	0.0704 (9)	
H59	0.2974	0.9751	0.5317	0.084*	
C60	0.3274 (4)	1.0482 (2)	0.45687 (18)	0.0828 (11)	
H60	0.2862	1.0912	0.4629	0.099*	
C61	0.3807 (4)	1.0538 (2)	0.40745 (17)	0.0867 (12)	
H61	0.3751	1.1004	0.3796	0.104*	
C62	0.4426 (4)	0.9904 (2)	0.39914 (15)	0.0927 (13)	
H62	0.4797	0.9945	0.3655	0.111*	
C63	0.4507 (4)	0.9209 (2)	0.43984 (14)	0.0780 (10)	
H63	0.4934	0.8784	0.4335	0.094*	
C64	−0.0076 (3)	0.64519 (19)	0.60137 (13)	0.0622 (9)	
C65	0.0684 (4)	0.7057 (2)	0.60501 (14)	0.0751 (10)	
H65	0.1556	0.6996	0.6039	0.090*	
C66	0.0184 (4)	0.7749 (2)	0.61019 (16)	0.0867 (12)	
H66	0.0718	0.8149	0.6121	0.104*	
C67	−0.1092 (5)	0.7849 (2)	0.61256 (18)	0.0971 (13)	
H67	−0.1432	0.8309	0.6171	0.116*	
C68	−0.1863 (4)	0.7270 (2)	0.60815 (19)	0.1032 (14)	
H68	−0.2733	0.7340	0.6090	0.124*	
C69	−0.1368 (4)	0.6580 (2)	0.60241 (16)	0.0827 (11)	
H69	−0.1911	0.6192	0.5992	0.099*	
C70	0.0463 (3)	0.56919 (18)	0.59860 (12)	0.0575 (8)	
C71	0.1609 (3)	0.5435 (2)	0.61956 (14)	0.0711 (10)	
H71	0.2045	0.5748	0.6351	0.085*	
C72	0.2118 (3)	0.4727 (2)	0.61792 (14)	0.0685 (9)	
H72	0.2893	0.4576	0.6320	0.082*	
C73	0.1497 (3)	0.42378 (18)	0.59562 (12)	0.0564 (8)	
C74	0.0361 (3)	0.44957 (19)	0.57408 (13)	0.0638 (9)	
H74	−0.0069	0.4185	0.5582	0.077*	
C75	−0.0149 (3)	0.52034 (19)	0.57562 (13)	0.0636 (9)	
H75	−0.0918	0.5358	0.5610	0.076*	
C76	0.2072 (3)	0.34779 (19)	0.59541 (13)	0.0599 (8)	
C77	0.1470 (3)	0.29011 (19)	0.58078 (14)	0.0666 (9)	
H77	0.0669	0.2996	0.5681	0.080*	
C78	0.2039 (3)	0.2175 (2)	0.58464 (14)	0.0679 (9)	
C79	0.1364 (3)	0.1508 (2)	0.57565 (14)	0.0655 (9)	
C80	0.1699 (4)	0.0748 (2)	0.60239 (15)	0.0791 (11)	
H80	0.2377	0.0664	0.6239	0.095*	
C81	0.1048 (5)	0.0114 (2)	0.59783 (17)	0.0919 (13)	
H81	0.1273	−0.0395	0.6168	0.110*	
C82	0.0065 (4)	0.0232 (3)	0.5654 (2)	0.0965 (14)	
H82	−0.0381	−0.0198	0.5623	0.116*	
C83	−0.0265 (4)	0.0987 (3)	0.5371 (2)	0.1031 (14)	
H83	−0.0922	0.1068	0.5145	0.124*	
C84	0.0386 (4)	0.1624 (2)	0.54246 (17)	0.0881 (12)	
H84	0.0162	0.2134	0.5235	0.106*	
C85	0.7565 (3)	−0.22780 (17)	0.09724 (12)	0.0546 (8)	
C86	0.8340 (3)	−0.2897 (2)	0.09348 (15)	0.0724 (10)	
H86	0.9211	−0.2821	0.0889	0.087*	
C87	0.7834 (4)	−0.3621 (2)	0.09647 (16)	0.0827 (11)	
H87	0.8365	−0.4029	0.0941	0.099*	
C88	0.6558 (4)	−0.3740 (2)	0.10284 (15)	0.0753 (10)	
H88	0.6220	−0.4228	0.1047	0.090*	
C89	0.5777 (3)	−0.3141 (2)	0.10649 (14)	0.0710 (10)	
H89	0.4907	−0.3223	0.1109	0.085*	
C90	0.6274 (3)	−0.24147 (19)	0.10365 (13)	0.0631 (9)	
H90	0.5733	−0.2011	0.1061	0.076*	
C91	0.8121 (3)	−0.15018 (17)	0.09457 (12)	0.0541 (8)	
C92	0.9297 (3)	−0.12523 (18)	0.06898 (13)	0.0599 (8)	
H92	0.9749	−0.1579	0.0530	0.072*	
C93	0.9814 (3)	−0.05386 (18)	0.06658 (13)	0.0619 (9)	
H93	1.0613	−0.0396	0.0497	0.074*	
C94	0.9167 (3)	−0.00299 (17)	0.08885 (12)	0.0525 (8)	
C95	0.7998 (3)	−0.02704 (18)	0.11531 (13)	0.0630 (9)	
H95	0.7554	0.0058	0.1314	0.076*	
C96	0.7484 (3)	−0.09917 (18)	0.11802 (13)	0.0632 (9)	
H96	0.6697	−0.1140	0.1359	0.076*	
C97	0.9728 (3)	0.07476 (19)	0.08389 (13)	0.0590 (8)	
C98	0.9081 (3)	0.13606 (18)	0.09470 (13)	0.0625 (9)	
H98	0.8248	0.1279	0.1088	0.075*	
C99	0.9644 (4)	0.2103 (2)	0.08496 (14)	0.0668 (9)	
C100	0.8937 (3)	0.27851 (19)	0.09247 (14)	0.0662 (9)	
C101	0.7873 (4)	0.2697 (2)	0.12713 (16)	0.0782 (11)	
H101	0.7595	0.2194	0.1473	0.094*	
C102	0.7220 (4)	0.3343 (2)	0.13221 (18)	0.0944 (13)	
H102	0.6510	0.3277	0.1559	0.113*	
C103	0.7623 (5)	0.4089 (3)	0.1020 (2)	0.1068 (15)	
H103	0.7178	0.4527	0.1051	0.128*	
C104	0.8675 (5)	0.4189 (2)	0.0675 (2)	0.1082 (15)	
H104	0.8944	0.4694	0.0472	0.130*	
C105	0.9333 (4)	0.3543 (2)	0.06286 (16)	0.0860 (12)	
H105	1.0053	0.3613	0.0396	0.103*	
C106	0.0752 (3)	−0.15222 (19)	0.25599 (13)	0.0562 (8)	
C107	0.0131 (3)	−0.1710 (2)	0.21710 (14)	0.0693 (9)	
H107	−0.0088	−0.1308	0.1872	0.083*	
C108	−0.0173 (4)	−0.2479 (2)	0.22154 (16)	0.0811 (11)	
H108	−0.0593	−0.2589	0.1948	0.097*	
C109	0.0138 (4)	−0.3083 (2)	0.26497 (19)	0.0856 (12)	
H109	−0.0065	−0.3602	0.2679	0.103*	
C110	0.0754 (4)	−0.2910 (2)	0.30415 (18)	0.0918 (13)	
H110	0.0975	−0.3316	0.3338	0.110*	
C111	0.1046 (3)	−0.2143 (2)	0.29990 (15)	0.0800 (11)	
H111	0.1451	−0.2037	0.3272	0.096*	
C112	0.1156 (3)	−0.07058 (19)	0.25050 (13)	0.0556 (8)	
C113	0.1400 (3)	−0.01567 (19)	0.20068 (14)	0.0616 (8)	
H113	0.1245	−0.0296	0.1705	0.074*	
C114	0.1862 (3)	0.0587 (2)	0.19444 (14)	0.0631 (9)	
H114	0.2022	0.0935	0.1603	0.076*	
C115	0.2094 (3)	0.0824 (2)	0.23827 (14)	0.0592 (8)	
C116	0.1825 (3)	0.0292 (2)	0.28834 (15)	0.0737 (10)	
H116	0.1962	0.0439	0.3184	0.088*	
C117	0.1356 (3)	−0.0453 (2)	0.29452 (14)	0.0684 (9)	
H117	0.1169	−0.0794	0.3287	0.082*	
C118	0.2652 (3)	0.1608 (2)	0.23286 (16)	0.0690 (9)	
C119	0.2692 (3)	0.2225 (2)	0.18547 (15)	0.0685 (9)	
H119	0.2310	0.2155	0.1565	0.082*	
C120	0.3283 (3)	0.2946 (2)	0.17956 (17)	0.0730 (10)	
C121	0.3364 (3)	0.3613 (2)	0.12977 (16)	0.0679 (9)	
C122	0.2953 (4)	0.3548 (2)	0.08281 (17)	0.0834 (11)	
H122	0.2627	0.3070	0.0819	0.100*	
C123	0.3021 (4)	0.4183 (3)	0.03741 (18)	0.1027 (14)	
H123	0.2742	0.4128	0.0061	0.123*	
C124	0.3493 (4)	0.4896 (3)	0.0375 (2)	0.0992 (13)	
H124	0.3534	0.5324	0.0065	0.119*	
C125	0.3899 (4)	0.4970 (2)	0.0834 (2)	0.0974 (14)	
H125	0.4218	0.5452	0.0838	0.117*	
C126	0.3846 (3)	0.4337 (2)	0.12953 (19)	0.0884 (12)	
H126	0.4134	0.4396	0.1606	0.106*	
O1	0.8195 (3)	0.25799 (15)	0.97179 (10)	0.0871 (8)	
H1A	0.857 (4)	0.325 (3)	0.9602 (17)	0.131*	
O2	0.8954 (2)	0.39522 (15)	0.92758 (11)	0.0836 (7)	
O3	0.6110 (2)	0.20682 (14)	0.74853 (9)	0.0714 (6)	
H3A	0.631 (3)	0.272 (2)	0.7511 (14)	0.107*	
O4	0.6002 (2)	0.34201 (14)	0.75623 (10)	0.0741 (7)	
O5	0.4111 (3)	0.70518 (15)	0.62114 (10)	0.0903 (8)	
H5A	0.363 (4)	0.777 (3)	0.6100 (17)	0.135*	
O6	0.3433 (3)	0.84226 (15)	0.57949 (10)	0.0863 (8)	
O7	0.3205 (2)	0.33673 (15)	0.61127 (11)	0.0833 (7)	
H7A	0.347 (4)	0.269 (3)	0.6064 (16)	0.125*	
O8	0.3175 (2)	0.20547 (15)	0.59834 (11)	0.0851 (7)	
O9	1.0918 (2)	0.08344 (15)	0.06661 (10)	0.0750 (7)	
H9A	1.109 (3)	0.152 (3)	0.0606 (14)	0.112*	
O10	1.0808 (3)	0.22160 (15)	0.06791 (11)	0.0878 (8)	
O11	0.3126 (3)	0.16920 (17)	0.27458 (11)	0.0952 (8)	
H11A	0.345 (4)	0.245 (3)	0.2566 (18)	0.143*	
O12	0.3820 (3)	0.30490 (17)	0.21993 (13)	0.1007 (9)	

### Atomic displacement parameters (Å^2^)

**Table:**

	*U*^11^	*U*^22^	*U*^33^	*U*^12^	*U*^13^	*U*^23^
C1	0.0436 (18)	0.061 (2)	0.065 (2)	0.0014 (15)	0.0072 (16)	−0.0211 (18)
C2	0.056 (2)	0.061 (2)	0.081 (2)	0.0003 (17)	−0.0077 (18)	−0.0231 (19)
C3	0.068 (2)	0.071 (3)	0.089 (3)	−0.0040 (19)	−0.011 (2)	−0.031 (2)
C4	0.081 (3)	0.064 (3)	0.098 (3)	−0.006 (2)	0.000 (2)	−0.031 (2)
C5	0.099 (3)	0.060 (2)	0.083 (3)	−0.002 (2)	−0.007 (2)	−0.014 (2)
C6	0.075 (2)	0.065 (2)	0.066 (2)	−0.0018 (19)	−0.0002 (18)	−0.0169 (19)
C7	0.0460 (18)	0.062 (2)	0.059 (2)	0.0012 (15)	0.0059 (15)	−0.0215 (18)
C8	0.064 (2)	0.065 (2)	0.059 (2)	−0.0029 (17)	−0.0030 (17)	−0.0237 (18)
C9	0.061 (2)	0.054 (2)	0.061 (2)	0.0006 (16)	−0.0005 (16)	−0.0128 (17)
C10	0.0492 (19)	0.061 (2)	0.064 (2)	0.0019 (15)	0.0015 (16)	−0.0241 (18)
C11	0.066 (2)	0.069 (2)	0.060 (2)	0.0010 (18)	0.0013 (17)	−0.0249 (19)
C12	0.067 (2)	0.064 (2)	0.057 (2)	−0.0033 (17)	0.0066 (17)	−0.0139 (17)
C13	0.053 (2)	0.067 (2)	0.072 (2)	0.0048 (17)	−0.0052 (17)	−0.029 (2)
C14	0.055 (2)	0.062 (2)	0.071 (2)	−0.0022 (16)	−0.0109 (17)	−0.0220 (19)
C15	0.053 (2)	0.064 (2)	0.085 (3)	0.0037 (17)	−0.0056 (18)	−0.030 (2)
C16	0.0484 (19)	0.058 (2)	0.089 (3)	0.0004 (16)	−0.0038 (18)	−0.026 (2)
C17	0.065 (2)	0.067 (2)	0.096 (3)	−0.0107 (19)	−0.005 (2)	−0.017 (2)
C18	0.082 (3)	0.087 (3)	0.099 (3)	−0.019 (2)	−0.012 (2)	−0.009 (3)
C19	0.077 (3)	0.069 (3)	0.122 (4)	−0.010 (2)	0.002 (3)	−0.001 (3)
C20	0.082 (3)	0.062 (3)	0.131 (4)	−0.008 (2)	0.007 (3)	−0.027 (3)
C21	0.070 (2)	0.068 (3)	0.109 (3)	−0.004 (2)	−0.003 (2)	−0.036 (2)
C22	0.062 (2)	0.056 (2)	0.0523 (19)	0.0034 (16)	−0.0018 (16)	−0.0172 (16)
C23	0.065 (2)	0.061 (2)	0.119 (3)	0.0034 (19)	−0.010 (2)	−0.029 (2)
C24	0.069 (3)	0.067 (3)	0.165 (4)	0.001 (2)	−0.023 (3)	−0.037 (3)
C25	0.098 (3)	0.061 (2)	0.099 (3)	−0.009 (2)	−0.023 (3)	−0.022 (2)
C26	0.104 (3)	0.063 (2)	0.089 (3)	−0.003 (2)	0.009 (2)	−0.037 (2)
C27	0.072 (2)	0.073 (2)	0.085 (3)	−0.004 (2)	0.012 (2)	−0.036 (2)
C28	0.053 (2)	0.058 (2)	0.0500 (19)	0.0029 (16)	−0.0018 (15)	−0.0201 (15)
C29	0.048 (2)	0.073 (2)	0.097 (3)	0.0004 (18)	−0.0002 (18)	−0.040 (2)
C30	0.053 (2)	0.063 (2)	0.093 (3)	0.0052 (17)	−0.0014 (18)	−0.038 (2)
C31	0.052 (2)	0.060 (2)	0.0499 (18)	0.0031 (16)	−0.0031 (15)	−0.0199 (15)
C32	0.0468 (19)	0.070 (2)	0.077 (2)	0.0050 (17)	−0.0113 (16)	−0.0301 (19)
C33	0.059 (2)	0.060 (2)	0.076 (2)	0.0110 (17)	−0.0099 (17)	−0.0301 (18)
C34	0.053 (2)	0.064 (2)	0.0447 (18)	−0.0018 (16)	−0.0005 (15)	−0.0172 (15)
C35	0.0499 (19)	0.065 (2)	0.068 (2)	−0.0016 (17)	−0.0031 (16)	−0.0264 (18)
C36	0.058 (2)	0.062 (2)	0.054 (2)	−0.0115 (17)	0.0001 (16)	−0.0197 (16)
C37	0.059 (2)	0.057 (2)	0.0504 (19)	−0.0034 (16)	−0.0026 (15)	−0.0195 (15)
C38	0.067 (2)	0.063 (2)	0.107 (3)	−0.0023 (19)	−0.012 (2)	−0.037 (2)
C39	0.072 (3)	0.082 (3)	0.127 (4)	0.008 (2)	−0.015 (2)	−0.048 (3)
C40	0.094 (3)	0.062 (2)	0.095 (3)	0.008 (2)	−0.020 (2)	−0.031 (2)
C41	0.101 (3)	0.056 (2)	0.078 (3)	−0.010 (2)	−0.016 (2)	−0.0263 (19)
C42	0.073 (2)	0.064 (2)	0.070 (2)	−0.0131 (19)	−0.0098 (18)	−0.0245 (18)
C43	0.0445 (18)	0.061 (2)	0.055 (2)	−0.0007 (15)	−0.0094 (15)	−0.0135 (16)
C44	0.056 (2)	0.062 (2)	0.062 (2)	0.0019 (16)	−0.0042 (16)	−0.0159 (18)
C45	0.072 (2)	0.065 (2)	0.068 (2)	−0.0041 (18)	−0.0132 (19)	−0.0089 (19)
C46	0.077 (3)	0.061 (2)	0.077 (3)	0.0049 (19)	−0.013 (2)	−0.021 (2)
C47	0.071 (2)	0.074 (3)	0.070 (2)	0.0074 (19)	−0.0018 (19)	−0.026 (2)
C48	0.063 (2)	0.064 (2)	0.061 (2)	0.0007 (17)	−0.0015 (17)	−0.0134 (17)
C49	0.0493 (19)	0.060 (2)	0.052 (2)	−0.0041 (15)	−0.0074 (15)	−0.0123 (17)
C50	0.080 (2)	0.062 (2)	0.051 (2)	−0.0015 (18)	−0.0052 (17)	−0.0062 (17)
C51	0.083 (3)	0.066 (2)	0.052 (2)	0.0007 (19)	0.0033 (18)	−0.0166 (18)
C52	0.059 (2)	0.060 (2)	0.053 (2)	−0.0043 (16)	−0.0029 (16)	−0.0140 (17)
C53	0.071 (2)	0.061 (2)	0.052 (2)	0.0008 (17)	−0.0098 (17)	−0.0084 (17)
C54	0.065 (2)	0.066 (2)	0.055 (2)	0.0013 (17)	−0.0022 (16)	−0.0189 (18)
C55	0.064 (2)	0.065 (2)	0.063 (2)	−0.0021 (17)	−0.0045 (18)	−0.0221 (19)
C56	0.066 (2)	0.064 (2)	0.058 (2)	0.0040 (17)	−0.0005 (17)	−0.0168 (18)
C57	0.061 (2)	0.070 (2)	0.065 (2)	−0.0046 (18)	0.0021 (18)	−0.0292 (19)
C58	0.0531 (19)	0.060 (2)	0.064 (2)	−0.0036 (16)	−0.0092 (16)	−0.0210 (18)
C59	0.069 (2)	0.069 (2)	0.076 (2)	0.0100 (19)	−0.0080 (19)	−0.027 (2)
C60	0.088 (3)	0.068 (3)	0.098 (3)	0.016 (2)	−0.023 (2)	−0.030 (2)
C61	0.100 (3)	0.075 (3)	0.080 (3)	0.014 (2)	−0.024 (2)	−0.014 (2)
C62	0.125 (4)	0.083 (3)	0.062 (3)	0.016 (3)	−0.004 (2)	−0.012 (2)
C63	0.098 (3)	0.065 (2)	0.069 (3)	0.009 (2)	0.000 (2)	−0.020 (2)
C64	0.068 (2)	0.056 (2)	0.062 (2)	−0.0013 (17)	−0.0076 (17)	−0.0170 (17)
C65	0.073 (2)	0.068 (2)	0.091 (3)	0.002 (2)	−0.018 (2)	−0.033 (2)
C66	0.093 (3)	0.063 (3)	0.112 (3)	0.001 (2)	−0.023 (3)	−0.036 (2)
C67	0.102 (4)	0.068 (3)	0.132 (4)	0.020 (2)	−0.028 (3)	−0.043 (3)
C68	0.082 (3)	0.081 (3)	0.157 (4)	0.023 (2)	−0.031 (3)	−0.047 (3)
C69	0.072 (3)	0.067 (3)	0.115 (3)	0.003 (2)	−0.022 (2)	−0.034 (2)
C70	0.054 (2)	0.059 (2)	0.058 (2)	−0.0075 (16)	−0.0053 (16)	−0.0165 (16)
C71	0.067 (2)	0.073 (2)	0.084 (3)	−0.0020 (19)	−0.018 (2)	−0.037 (2)
C72	0.060 (2)	0.072 (2)	0.080 (2)	0.0055 (18)	−0.0227 (18)	−0.029 (2)
C73	0.0509 (19)	0.059 (2)	0.060 (2)	−0.0048 (16)	−0.0032 (16)	−0.0178 (16)
C74	0.060 (2)	0.059 (2)	0.075 (2)	−0.0080 (17)	−0.0123 (18)	−0.0219 (18)
C75	0.052 (2)	0.060 (2)	0.082 (2)	0.0000 (16)	−0.0160 (17)	−0.0236 (18)
C76	0.053 (2)	0.064 (2)	0.061 (2)	−0.0011 (17)	−0.0046 (16)	−0.0171 (17)
C77	0.053 (2)	0.066 (2)	0.086 (3)	0.0055 (17)	−0.0087 (18)	−0.031 (2)
C78	0.058 (2)	0.076 (3)	0.072 (2)	0.0034 (19)	−0.0022 (18)	−0.0269 (19)
C79	0.067 (2)	0.058 (2)	0.074 (2)	0.0038 (18)	0.0044 (19)	−0.0260 (19)
C80	0.097 (3)	0.065 (3)	0.078 (3)	0.004 (2)	−0.001 (2)	−0.027 (2)
C81	0.123 (4)	0.066 (3)	0.087 (3)	−0.003 (3)	0.009 (3)	−0.029 (2)
C82	0.101 (3)	0.077 (3)	0.126 (4)	−0.016 (3)	0.022 (3)	−0.057 (3)
C83	0.088 (3)	0.088 (3)	0.153 (4)	0.004 (3)	−0.023 (3)	−0.063 (3)
C84	0.081 (3)	0.072 (3)	0.122 (4)	0.011 (2)	−0.021 (3)	−0.043 (2)
C85	0.059 (2)	0.0496 (19)	0.059 (2)	0.0033 (15)	−0.0113 (16)	−0.0195 (15)
C86	0.055 (2)	0.064 (2)	0.108 (3)	0.0037 (17)	−0.0126 (19)	−0.039 (2)
C87	0.075 (3)	0.057 (2)	0.128 (3)	0.0077 (19)	−0.013 (2)	−0.045 (2)
C88	0.075 (3)	0.057 (2)	0.098 (3)	−0.0052 (19)	−0.013 (2)	−0.029 (2)
C89	0.061 (2)	0.066 (2)	0.087 (3)	−0.0089 (19)	−0.0056 (19)	−0.024 (2)
C90	0.059 (2)	0.058 (2)	0.075 (2)	0.0031 (17)	−0.0055 (17)	−0.0244 (18)
C91	0.053 (2)	0.0501 (19)	0.059 (2)	0.0047 (15)	−0.0077 (16)	−0.0164 (16)
C92	0.061 (2)	0.056 (2)	0.069 (2)	0.0076 (16)	−0.0012 (17)	−0.0282 (17)
C93	0.057 (2)	0.059 (2)	0.072 (2)	−0.0007 (16)	0.0008 (17)	−0.0253 (18)
C94	0.0493 (19)	0.0483 (18)	0.062 (2)	0.0015 (15)	−0.0066 (15)	−0.0184 (16)
C95	0.064 (2)	0.053 (2)	0.077 (2)	0.0032 (17)	0.0024 (18)	−0.0292 (18)
C96	0.053 (2)	0.061 (2)	0.079 (2)	−0.0011 (16)	0.0064 (17)	−0.0284 (18)
C97	0.056 (2)	0.061 (2)	0.064 (2)	0.0019 (17)	−0.0113 (17)	−0.0227 (17)
C98	0.056 (2)	0.055 (2)	0.079 (2)	0.0007 (16)	−0.0056 (17)	−0.0253 (18)
C99	0.069 (2)	0.059 (2)	0.080 (2)	−0.0040 (19)	−0.015 (2)	−0.0297 (19)
C100	0.075 (2)	0.054 (2)	0.076 (2)	−0.0007 (18)	−0.026 (2)	−0.0258 (19)
C101	0.087 (3)	0.060 (2)	0.094 (3)	−0.001 (2)	−0.014 (2)	−0.030 (2)
C102	0.103 (3)	0.077 (3)	0.117 (4)	0.018 (3)	−0.016 (3)	−0.048 (3)
C103	0.135 (4)	0.066 (3)	0.133 (4)	0.027 (3)	−0.037 (3)	−0.045 (3)
C104	0.144 (5)	0.056 (3)	0.127 (4)	0.008 (3)	−0.034 (4)	−0.027 (3)
C105	0.106 (3)	0.058 (2)	0.096 (3)	−0.003 (2)	−0.022 (2)	−0.022 (2)
C106	0.0381 (17)	0.066 (2)	0.062 (2)	0.0002 (15)	0.0003 (15)	−0.0163 (18)
C107	0.065 (2)	0.066 (2)	0.075 (2)	0.0034 (18)	−0.0113 (19)	−0.0184 (19)
C108	0.082 (3)	0.071 (3)	0.090 (3)	−0.008 (2)	−0.011 (2)	−0.023 (2)
C109	0.070 (3)	0.067 (3)	0.115 (4)	−0.010 (2)	0.006 (2)	−0.024 (3)
C110	0.080 (3)	0.073 (3)	0.101 (3)	−0.012 (2)	−0.014 (2)	0.007 (2)
C111	0.069 (2)	0.084 (3)	0.074 (3)	−0.012 (2)	−0.007 (2)	−0.004 (2)
C112	0.0372 (17)	0.067 (2)	0.062 (2)	0.0049 (15)	−0.0038 (15)	−0.0192 (18)
C113	0.056 (2)	0.066 (2)	0.066 (2)	0.0043 (17)	−0.0096 (17)	−0.0254 (19)
C114	0.060 (2)	0.065 (2)	0.065 (2)	0.0043 (17)	−0.0064 (17)	−0.0217 (18)
C115	0.0479 (19)	0.067 (2)	0.069 (2)	0.0093 (16)	−0.0071 (16)	−0.0292 (19)
C116	0.071 (2)	0.090 (3)	0.070 (3)	0.000 (2)	−0.0104 (19)	−0.038 (2)
C117	0.063 (2)	0.081 (3)	0.060 (2)	−0.0004 (19)	−0.0060 (17)	−0.0192 (19)
C118	0.053 (2)	0.080 (3)	0.085 (3)	0.0089 (18)	−0.0155 (19)	−0.039 (2)
C119	0.061 (2)	0.070 (2)	0.084 (3)	−0.0013 (18)	−0.0140 (19)	−0.034 (2)
C120	0.057 (2)	0.078 (3)	0.100 (3)	0.0080 (19)	−0.021 (2)	−0.048 (2)
C121	0.053 (2)	0.062 (2)	0.096 (3)	0.0003 (17)	−0.0075 (19)	−0.034 (2)
C122	0.096 (3)	0.070 (3)	0.095 (3)	−0.007 (2)	−0.004 (2)	−0.041 (2)
C123	0.133 (4)	0.085 (3)	0.092 (3)	−0.017 (3)	−0.005 (3)	−0.028 (3)
C124	0.085 (3)	0.090 (3)	0.120 (4)	−0.015 (2)	−0.003 (3)	−0.028 (3)
C125	0.074 (3)	0.067 (3)	0.151 (4)	−0.011 (2)	−0.022 (3)	−0.029 (3)
C126	0.073 (3)	0.079 (3)	0.126 (4)	−0.002 (2)	−0.030 (2)	−0.043 (3)
O1	0.115 (2)	0.0733 (17)	0.0788 (18)	−0.0072 (15)	−0.0237 (16)	−0.0285 (14)
O2	0.0937 (19)	0.0717 (17)	0.094 (2)	−0.0030 (14)	−0.0219 (16)	−0.0346 (15)
O3	0.0497 (14)	0.0723 (16)	0.0941 (18)	−0.0024 (12)	−0.0069 (12)	−0.0277 (14)
O4	0.0568 (15)	0.0720 (16)	0.0970 (18)	−0.0068 (12)	−0.0079 (13)	−0.0301 (14)
O5	0.127 (2)	0.0739 (17)	0.0641 (16)	0.0133 (16)	0.0173 (15)	−0.0177 (13)
O6	0.106 (2)	0.0730 (17)	0.0753 (17)	0.0084 (15)	0.0215 (15)	−0.0215 (14)
O7	0.0647 (16)	0.0781 (17)	0.115 (2)	0.0099 (13)	−0.0272 (15)	−0.0376 (16)
O8	0.0710 (18)	0.0760 (18)	0.114 (2)	0.0137 (14)	−0.0186 (15)	−0.0355 (16)
O9	0.0578 (15)	0.0729 (16)	0.0995 (19)	−0.0068 (12)	0.0041 (13)	−0.0359 (14)
O10	0.0745 (18)	0.0729 (17)	0.122 (2)	−0.0160 (14)	0.0023 (16)	−0.0406 (16)
O11	0.113 (2)	0.090 (2)	0.093 (2)	−0.0050 (16)	−0.0384 (17)	−0.0355 (16)
O12	0.111 (2)	0.086 (2)	0.117 (2)	−0.0121 (16)	−0.0431 (19)	−0.0391 (18)

### Geometric parameters (Å, °)

**Table:**

C1—C2	1.382 (4)	C65—H65	0.9300
C1—C6	1.386 (4)	C66—C67	1.363 (5)
C1—C7	1.486 (4)	C66—H66	0.9300
C2—C3	1.374 (4)	C67—C68	1.360 (5)
C2—H2	0.9300	C67—H67	0.9300
C3—C4	1.375 (5)	C68—C69	1.379 (5)
C3—H3	0.9300	C68—H68	0.9300
C4—C5	1.368 (5)	C69—H69	0.9300
C4—H4	0.9300	C70—C71	1.385 (4)
C5—C6	1.382 (5)	C70—C75	1.391 (4)
C5—H5	0.9300	C71—C72	1.379 (4)
C6—H6	0.9300	C71—H71	0.9300
C7—C8	1.387 (4)	C72—C73	1.385 (4)
C7—C12	1.389 (4)	C72—H72	0.9300
C8—C9	1.378 (4)	C73—C74	1.382 (4)
C8—H8	0.9300	C73—C76	1.481 (4)
C9—C10	1.380 (4)	C74—C75	1.378 (4)
C9—H9	0.9300	C74—H74	0.9300
C10—C11	1.392 (4)	C75—H75	0.9300
C10—C13	1.479 (4)	C76—O7	1.293 (4)
C11—C12	1.382 (4)	C76—C77	1.379 (4)
C11—H11	0.9300	C77—C78	1.399 (4)
C12—H12	0.9300	C77—H77	0.9300
C13—O1	1.294 (4)	C78—O8	1.280 (4)
C13—C14	1.390 (4)	C78—C79	1.481 (5)
C14—C15	1.393 (4)	C79—C84	1.377 (5)
C14—H14	0.9300	C79—C80	1.378 (5)
C15—O2	1.288 (4)	C80—C81	1.369 (5)
C15—C16	1.479 (5)	C80—H80	0.9300
C16—C17	1.383 (5)	C81—C82	1.369 (6)
C16—C21	1.386 (4)	C81—H81	0.9300
C17—C18	1.379 (5)	C82—C83	1.379 (6)
C17—H17	0.9300	C82—H82	0.9300
C18—C19	1.372 (5)	C83—C84	1.383 (5)
C18—H18	0.9300	C83—H83	0.9300
C19—C20	1.373 (6)	C84—H84	0.9300
C19—H19	0.9300	C85—C90	1.383 (4)
C20—C21	1.371 (5)	C85—C86	1.392 (4)
C20—H20	0.9300	C85—C91	1.488 (4)
C21—H21	0.9300	C86—C87	1.379 (4)
C22—C23	1.381 (4)	C86—H86	0.9300
C22—C27	1.381 (4)	C87—C88	1.362 (5)
C22—C28	1.491 (4)	C87—H87	0.9300
C23—C24	1.377 (5)	C88—C89	1.368 (4)
C23—H23	0.9300	C88—H88	0.9300
C24—C25	1.362 (5)	C89—C90	1.380 (4)
C24—H24	0.9300	C89—H89	0.9300
C25—C26	1.365 (5)	C90—H90	0.9300
C25—H25	0.9300	C91—C92	1.387 (4)
C26—C27	1.383 (5)	C91—C96	1.393 (4)
C26—H26	0.9300	C92—C93	1.372 (4)
C27—H27	0.9300	C92—H92	0.9300
C28—C33	1.381 (4)	C93—C94	1.377 (4)
C28—C29	1.388 (4)	C93—H93	0.9300
C29—C30	1.379 (4)	C94—C95	1.385 (4)
C29—H29	0.9300	C94—C97	1.478 (4)
C30—C31	1.382 (4)	C95—C96	1.380 (4)
C30—H30	0.9300	C95—H95	0.9300
C31—C32	1.382 (4)	C96—H96	0.9300
C31—C34	1.477 (4)	C97—O9	1.307 (4)
C32—C33	1.374 (4)	C97—C98	1.376 (4)
C32—H32	0.9300	C98—C99	1.400 (4)
C33—H33	0.9300	C98—H98	0.9300
C34—O3	1.304 (3)	C99—O10	1.281 (4)
C34—C35	1.379 (4)	C99—C100	1.479 (5)
C35—C36	1.409 (4)	C100—C101	1.382 (5)
C35—H35	0.9300	C100—C105	1.388 (5)
C36—O4	1.271 (4)	C101—C102	1.374 (5)
C36—C37	1.482 (4)	C101—H101	0.9300
C37—C38	1.374 (4)	C102—C103	1.379 (6)
C37—C42	1.385 (3)	C102—H102	0.9300
C38—C39	1.379 (5)	C103—C104	1.368 (6)
C38—H38	0.9300	C103—H103	0.9300
C39—C40	1.368 (5)	C104—C105	1.374 (5)
C39—H39	0.9300	C104—H104	0.9300
C40—C41	1.369 (5)	C105—H105	0.9300
C40—H40	0.9300	C106—C107	1.381 (4)
C41—C42	1.380 (4)	C106—C111	1.386 (4)
C41—H41	0.9300	C106—C112	1.481 (4)
C42—H42	0.9300	C107—C108	1.379 (5)
C43—C48	1.388	C107—H107	0.9300
C43—C44	1.3890	C108—C109	1.367 (5)
C43—C49	1.486	C108—H108	0.9300
C44—C45	1.381	C109—C110	1.372 (5)
C44—H44	0.9300	C109—H109	0.9300
C45—C46	1.375 (5)	C110—C111	1.374 (5)
C45—H45	0.9300	C110—H110	0.9300
C46—C47	1.373 (5)	C111—H111	0.9300
C46—H46	0.9300	C112—C113	1.387 (4)
C47—C48	1.381 (4)	C112—C117	1.398 (4)
C47—H47	0.9300	C113—C114	1.375 (4)
C48—H48	0.9300	C113—H113	0.9300
C49—C54	1.383 (4)	C114—C115	1.385 (4)
C49—C50	1.384 (4)	C114—H114	0.9300
C50—C51	1.381 (4)	C115—C116	1.383 (5)
C50—H50	0.9300	C115—C118	1.484 (5)
C51—C52	1.381 (4)	C116—C117	1.379 (4)
C51—H51	0.9300	C116—H116	0.9300
C52—C53	1.385 (4)	C117—H117	0.9300
C52—C55	1.482 (4)	C118—O11	1.291 (4)
C53—C54	1.377 (4)	C118—C119	1.388 (5)
C53—H53	0.9300	C119—C120	1.396 (5)
C54—H54	0.9300	C119—H119	0.9300
C55—O5	1.283 (4)	C120—O12	1.303 (4)
C55—C56	1.402 (4)	C120—C121	1.475 (5)
C56—C57	1.377 (4)	C121—C122	1.379 (5)
C56—H56	0.9300	C121—C126	1.390 (5)
C57—O6	1.300 (4)	C122—C123	1.371 (5)
C57—C58	1.477 (4)	C122—H122	0.9300
C58—C63	1.378 (4)	C123—C124	1.371 (5)
C58—C59	1.384 (4)	C123—H123	0.9300
C59—C60	1.382 (5)	C124—C125	1.358 (6)
C59—H59	0.9300	C124—H124	0.9300
C60—C61	1.360 (5)	C125—C126	1.381 (5)
C60—H60	0.9300	C125—H125	0.9300
C61—C62	1.368 (5)	C126—H126	0.9300
C61—H61	0.9300	O1—H1A	1.21 (5)
C62—C63	1.376 (5)	O3—H3A	1.20 (4)
C62—H62	0.9300	O6—H5A	1.22 (5)
C63—H63	0.9300	O7—H7A	1.27 (5)
C64—C65	1.383 (4)	O8—H7A	1.26 (4)
C64—C69	1.387 (5)	O9—H9A	1.19 (4)
C64—C70	1.485 (4)	O12—H11A	1.24 (5)
C65—C66	1.378 (5)		
			
C2—C1—C6	117.7 (3)	C67—C66—C65	120.2 (4)
C2—C1—C7	121.1 (3)	C67—C66—H66	119.9
C6—C1—C7	121.2 (3)	C65—C66—H66	119.9
C3—C2—C1	121.3 (3)	C68—C67—C66	119.4 (4)
C3—C2—H2	119.4	C68—C67—H67	120.3
C1—C2—H2	119.4	C66—C67—H67	120.3
C2—C3—C4	120.4 (4)	C67—C68—C69	120.7 (4)
C2—C3—H3	119.8	C67—C68—H68	119.6
C4—C3—H3	119.8	C69—C68—H68	119.6
C5—C4—C3	119.2 (4)	C68—C69—C64	121.2 (4)
C5—C4—H4	120.4	C68—C69—H69	119.4
C3—C4—H4	120.4	C64—C69—H69	119.4
C4—C5—C6	120.5 (4)	C71—C70—C75	116.9 (3)
C4—C5—H5	119.7	C71—C70—C64	120.8 (3)
C6—C5—H5	119.7	C75—C70—C64	122.4 (3)
C5—C6—C1	120.9 (3)	C72—C71—C70	121.6 (3)
C5—C6—H6	119.6	C72—C71—H71	119.2
C1—C6—H6	119.6	C70—C71—H71	119.2
C8—C7—C12	116.9 (3)	C71—C72—C73	121.3 (3)
C8—C7—C1	121.2 (3)	C71—C72—H72	119.4
C12—C7—C1	121.9 (3)	C73—C72—H72	119.4
C9—C8—C7	121.6 (3)	C74—C73—C72	117.3 (3)
C9—C8—H8	119.2	C74—C73—C76	123.3 (3)
C7—C8—H8	119.2	C72—C73—C76	119.4 (3)
C8—C9—C10	121.5 (3)	C75—C74—C73	121.5 (3)
C8—C9—H9	119.3	C75—C74—H74	119.3
C10—C9—H9	119.3	C73—C74—H74	119.3
C9—C10—C11	117.5 (3)	C74—C75—C70	121.4 (3)
C9—C10—C13	122.5 (3)	C74—C75—H75	119.3
C11—C10—C13	120.0 (3)	C70—C75—H75	119.3
C12—C11—C10	120.9 (3)	O7—C76—C77	120.2 (3)
C12—C11—H11	119.5	O7—C76—C73	115.5 (3)
C10—C11—H11	119.5	C77—C76—C73	124.3 (3)
C11—C12—C7	121.6 (3)	C76—C77—C78	121.4 (3)
C11—C12—H12	119.2	C76—C77—H77	119.3
C7—C12—H12	119.2	C78—C77—H77	119.3
O1—C13—C14	120.3 (3)	O8—C78—C77	120.2 (3)
O1—C13—C10	116.4 (3)	O8—C78—C79	116.9 (3)
C14—C13—C10	123.3 (3)	C77—C78—C79	122.9 (3)
C13—C14—C15	121.5 (3)	C84—C79—C80	118.8 (3)
C13—C14—H14	119.2	C84—C79—C78	121.9 (3)
C15—C14—H14	119.2	C80—C79—C78	119.2 (4)
O2—C15—C14	120.4 (3)	C81—C80—C79	121.1 (4)
O2—C15—C16	116.2 (3)	C81—C80—H80	119.4
C14—C15—C16	123.4 (3)	C79—C80—H80	119.4
C17—C16—C21	118.2 (4)	C82—C81—C80	119.9 (4)
C17—C16—C15	122.2 (3)	C82—C81—H81	120.1
C21—C16—C15	119.6 (4)	C80—C81—H81	120.1
C18—C17—C16	120.4 (4)	C81—C82—C83	120.0 (4)
C18—C17—H17	119.8	C81—C82—H82	120.0
C16—C17—H17	119.8	C83—C82—H82	120.0
C19—C18—C17	120.7 (4)	C82—C83—C84	119.8 (4)
C19—C18—H18	119.6	C82—C83—H83	120.1
C17—C18—H18	119.6	C84—C83—H83	120.1
C18—C19—C20	119.1 (4)	C79—C84—C83	120.4 (4)
C18—C19—H19	120.5	C79—C84—H84	119.8
C20—C19—H19	120.5	C83—C84—H84	119.8
C21—C20—C19	120.6 (4)	C90—C85—C86	117.6 (3)
C21—C20—H20	119.7	C90—C85—C91	121.8 (3)
C19—C20—H20	119.7	C86—C85—C91	120.6 (3)
C20—C21—C16	120.9 (4)	C87—C86—C85	121.0 (3)
C20—C21—H21	119.5	C87—C86—H86	119.5
C16—C21—H21	119.5	C85—C86—H86	119.5
C23—C22—C27	116.6 (3)	C88—C87—C86	120.3 (3)
C23—C22—C28	122.2 (3)	C88—C87—H87	119.9
C27—C22—C28	121.2 (3)	C86—C87—H87	119.9
C24—C23—C22	121.3 (4)	C87—C88—C89	119.8 (3)
C24—C23—H23	119.4	C87—C88—H88	120.1
C22—C23—H23	119.4	C89—C88—H88	120.1
C25—C24—C23	120.8 (4)	C88—C89—C90	120.4 (3)
C25—C24—H24	119.6	C88—C89—H89	119.8
C23—C24—H24	119.6	C90—C89—H89	119.8
C24—C25—C26	119.6 (4)	C89—C90—C85	121.0 (3)
C24—C25—H25	120.2	C89—C90—H90	119.5
C26—C25—H25	120.2	C85—C90—H90	119.5
C25—C26—C27	119.3 (4)	C92—C91—C96	116.7 (3)
C25—C26—H26	120.3	C92—C91—C85	121.8 (3)
C27—C26—H26	120.3	C96—C91—C85	121.5 (3)
C22—C27—C26	122.4 (4)	C93—C92—C91	122.0 (3)
C22—C27—H27	118.8	C93—C92—H92	119.0
C26—C27—H27	118.8	C91—C92—H92	119.0
C33—C28—C29	116.3 (3)	C92—C93—C94	120.9 (3)
C33—C28—C22	122.0 (3)	C92—C93—H93	119.5
C29—C28—C22	121.7 (3)	C94—C93—H93	119.5
C30—C29—C28	122.0 (3)	C93—C94—C95	118.1 (3)
C30—C29—H29	119.0	C93—C94—C97	119.7 (3)
C28—C29—H29	119.0	C95—C94—C97	122.1 (3)
C29—C30—C31	120.9 (3)	C96—C95—C94	120.8 (3)
C29—C30—H30	119.5	C96—C95—H95	119.6
C31—C30—H30	119.5	C94—C95—H95	119.6
C32—C31—C30	117.5 (3)	C95—C96—C91	121.4 (3)
C32—C31—C34	119.8 (3)	C95—C96—H96	119.3
C30—C31—C34	122.6 (3)	C91—C96—H96	119.3
C33—C32—C31	121.0 (3)	O9—C97—C98	120.3 (3)
C33—C32—H32	119.5	O9—C97—C94	114.9 (3)
C31—C32—H32	119.5	C98—C97—C94	124.7 (3)
C32—C33—C28	122.3 (3)	C97—C98—C99	121.7 (3)
C32—C33—H33	118.8	C97—C98—H98	119.2
C28—C33—H33	118.8	C99—C98—H98	119.2
O3—C34—C35	120.2 (3)	O10—C99—C98	120.1 (3)
O3—C34—C31	115.0 (3)	O10—C99—C100	117.5 (3)
C35—C34—C31	124.8 (3)	C98—C99—C100	122.5 (3)
C34—C35—C36	121.8 (3)	C101—C100—C105	118.4 (3)
C34—C35—H35	119.1	C101—C100—C99	122.4 (3)
C36—C35—H35	119.1	C105—C100—C99	119.2 (4)
O4—C36—C35	119.8 (3)	C102—C101—C100	120.9 (4)
O4—C36—C37	117.8 (3)	C102—C101—H101	119.6
C35—C36—C37	122.3 (3)	C100—C101—H101	119.6
C38—C37—C42	117.9 (3)	C101—C102—C103	119.7 (4)
C38—C37—C36	122.6 (3)	C101—C102—H102	120.2
C42—C37—C36	119.5 (3)	C103—C102—H102	120.2
C37—C38—C39	121.5 (3)	C104—C103—C102	120.3 (4)
C37—C38—H38	119.2	C104—C103—H103	119.8
C39—C38—H38	119.2	C102—C103—H103	119.8
C40—C39—C38	119.9 (4)	C103—C104—C105	119.9 (4)
C40—C39—H39	120.0	C103—C104—H104	120.1
C38—C39—H39	120.0	C105—C104—H104	120.1
C39—C40—C41	119.6 (4)	C104—C105—C100	120.8 (4)
C39—C40—H40	120.2	C104—C105—H105	119.6
C41—C40—H40	120.2	C100—C105—H105	119.6
C40—C41—C42	120.4 (3)	C107—C106—C111	116.9 (3)
C40—C41—H41	119.8	C107—C106—C112	122.4 (3)
C42—C41—H41	119.8	C111—C106—C112	120.7 (3)
C41—C42—C37	120.7 (3)	C108—C107—C106	121.5 (3)
C41—C42—H42	119.7	C108—C107—H107	119.2
C37—C42—H42	119.7	C106—C107—H107	119.2
C48—C43—C44	116.86	C109—C108—C107	120.6 (4)
C48—C43—C49	121.8	C109—C108—H108	119.7
C44—C43—C49	121.30	C107—C108—H108	119.7
C45—C44—C43	121.73	C108—C109—C110	118.9 (4)
C45—C44—H44	119.2	C108—C109—H109	120.5
C43—C44—H44	119.1	C110—C109—H109	120.5
C46—C45—C44	120.3	C109—C110—C111	120.5 (4)
C46—C45—H45	119.9	C109—C110—H110	119.8
C44—C45—H45	119.9	C111—C110—H110	119.8
C47—C46—C45	119.0 (3)	C110—C111—C106	121.6 (4)
C47—C46—H46	120.5	C110—C111—H111	119.2
C45—C46—H46	120.5	C106—C111—H111	119.2
C46—C47—C48	120.7 (3)	C113—C112—C117	116.4 (3)
C46—C47—H47	119.7	C113—C112—C106	121.1 (3)
C48—C47—H47	119.7	C117—C112—C106	122.5 (3)
C47—C48—C43	121.4	C114—C113—C112	122.2 (3)
C47—C48—H48	119.3	C114—C113—H113	118.9
C43—C48—H48	119.3	C112—C113—H113	118.9
C54—C49—C50	117.2 (3)	C113—C114—C115	121.0 (3)
C54—C49—C43	121.4	C113—C114—H114	119.5
C50—C49—C43	121.3	C115—C114—H114	119.5
C51—C50—C49	121.4 (3)	C116—C115—C114	117.6 (3)
C51—C50—H50	119.3	C116—C115—C118	120.1 (3)
C49—C50—H50	119.3	C114—C115—C118	122.2 (3)
C52—C51—C50	120.9 (3)	C117—C116—C115	121.3 (3)
C52—C51—H51	119.6	C117—C116—H116	119.3
C50—C51—H51	119.6	C115—C116—H116	119.3
C51—C52—C53	118.1 (3)	C116—C117—C112	121.4 (3)
C51—C52—C55	119.4 (3)	C116—C117—H117	119.3
C53—C52—C55	122.5 (3)	C112—C117—H117	119.3
C54—C53—C52	120.6 (3)	O11—C118—C119	120.6 (3)
C54—C53—H53	119.7	O11—C118—C115	116.7 (4)
C52—C53—H53	119.7	C119—C118—C115	122.7 (3)
C53—C54—C49	121.8 (3)	C118—C119—C120	122.6 (3)
C53—C54—H54	119.1	C118—C119—H119	118.7
C49—C54—H54	119.1	C120—C119—H119	118.7
O5—C55—C56	120.3 (3)	O12—C120—C119	119.1 (4)
O5—C55—C52	116.4 (3)	O12—C120—C121	116.6 (3)
C56—C55—C52	123.3 (3)	C119—C120—C121	124.3 (3)
C57—C56—C55	121.4 (3)	C122—C121—C126	118.1 (4)
C57—C56—H56	119.3	C122—C121—C120	122.0 (3)
C55—C56—H56	119.3	C126—C121—C120	119.9 (4)
O6—C57—C56	120.4 (3)	C123—C122—C121	120.6 (4)
O6—C57—C58	115.3 (3)	C123—C122—H122	119.7
C56—C57—C58	124.3 (3)	C121—C122—H122	119.7
C63—C58—C59	118.4 (3)	C122—C123—C124	121.1 (4)
C63—C58—C57	122.3 (3)	C122—C123—H123	119.5
C59—C58—C57	119.3 (3)	C124—C123—H123	119.5
C60—C59—C58	120.7 (4)	C125—C124—C123	119.0 (5)
C60—C59—H59	119.6	C125—C124—H124	120.5
C58—C59—H59	119.6	C123—C124—H124	120.5
C61—C60—C59	120.2 (4)	C124—C125—C126	120.8 (4)
C61—C60—H60	119.9	C124—C125—H125	119.6
C59—C60—H60	119.9	C126—C125—H125	119.6
C60—C61—C62	119.4 (4)	C125—C126—C121	120.4 (4)
C60—C61—H61	120.3	C125—C126—H126	119.8
C62—C61—H61	120.3	C121—C126—H126	119.8
C61—C62—C63	121.0 (4)	C13—O1—H1A	101 (2)
C61—C62—H62	119.5	C15—O2—H1A	99.8 (18)
C63—C62—H62	119.5	C34—O3—H3A	101.3 (17)
C62—C63—C58	120.2 (4)	C36—O4—H3A	101.2 (16)
C62—C63—H63	119.9	C55—O5—H5A	102.3 (18)
C58—C63—H63	119.9	C57—O6—H5A	103 (2)
C65—C64—C69	116.7 (3)	C76—O7—H7A	102.5 (18)
C65—C64—C70	121.5 (3)	C78—O8—H7A	102.8 (18)
C69—C64—C70	121.7 (3)	C97—O9—H9A	101.3 (18)
C66—C65—C64	121.7 (4)	C99—O10—H9A	100.7 (16)
C66—C65—H65	119.1	C118—O11—H11A	100.3 (19)
C64—C65—H65	119.1	C120—O12—H11A	102 (2)
			
C6—C1—C2—C3	0.4 (5)	C69—C64—C65—C66	0.9 (5)
C7—C1—C2—C3	179.1 (3)	C70—C64—C65—C66	−176.8 (3)
C1—C2—C3—C4	−0.1 (5)	C64—C65—C66—C67	0.9 (6)
C2—C3—C4—C5	−0.2 (6)	C65—C66—C67—C68	−2.0 (7)
C3—C4—C5—C6	0.2 (6)	C66—C67—C68—C69	1.3 (7)
C4—C5—C6—C1	0.2 (6)	C67—C68—C69—C64	0.5 (7)
C2—C1—C6—C5	−0.5 (5)	C65—C64—C69—C68	−1.6 (6)
C7—C1—C6—C5	−179.1 (3)	C70—C64—C69—C68	176.1 (4)
C2—C1—C7—C8	−27.5 (4)	C65—C64—C70—C71	25.2 (5)
C6—C1—C7—C8	151.1 (3)	C69—C64—C70—C71	−152.4 (4)
C2—C1—C7—C12	153.2 (3)	C65—C64—C70—C75	−155.3 (3)
C6—C1—C7—C12	−28.1 (4)	C69—C64—C70—C75	27.1 (5)
C12—C7—C8—C9	1.1 (5)	C75—C70—C71—C72	−0.2 (5)
C1—C7—C8—C9	−178.2 (3)	C64—C70—C71—C72	179.3 (3)
C7—C8—C9—C10	−0.7 (5)	C70—C71—C72—C73	−0.6 (6)
C8—C9—C10—C11	−0.3 (5)	C71—C72—C73—C74	1.4 (5)
C8—C9—C10—C13	177.7 (3)	C71—C72—C73—C76	−179.2 (3)
C9—C10—C11—C12	0.9 (5)	C72—C73—C74—C75	−1.3 (5)
C13—C10—C11—C12	−177.1 (3)	C76—C73—C74—C75	179.3 (3)
C10—C11—C12—C7	−0.5 (5)	C73—C74—C75—C70	0.5 (5)
C8—C7—C12—C11	−0.4 (5)	C71—C70—C75—C74	0.3 (5)
C1—C7—C12—C11	178.8 (3)	C64—C70—C75—C74	−179.2 (3)
C9—C10—C13—O1	−170.7 (3)	C74—C73—C76—O7	172.0 (3)
C11—C10—C13—O1	7.3 (4)	C72—C73—C76—O7	−7.4 (5)
C9—C10—C13—C14	7.8 (5)	C74—C73—C76—C77	−9.4 (5)
C11—C10—C13—C14	−174.3 (3)	C72—C73—C76—C77	171.2 (3)
O1—C13—C14—C15	1.1 (5)	O7—C76—C77—C78	2.6 (5)
C10—C13—C14—C15	−177.2 (3)	C73—C76—C77—C78	−176.0 (3)
C13—C14—C15—O2	0.1 (5)	C76—C77—C78—O8	−5.1 (5)
C13—C14—C15—C16	−179.7 (3)	C76—C77—C78—C79	172.3 (3)
O2—C15—C16—C17	179.5 (3)	O8—C78—C79—C84	−154.5 (4)
C14—C15—C16—C17	−0.7 (5)	C77—C78—C79—C84	28.1 (5)
O2—C15—C16—C21	−1.7 (5)	O8—C78—C79—C80	27.5 (5)
C14—C15—C16—C21	178.1 (3)	C77—C78—C79—C80	−150.0 (3)
C21—C16—C17—C18	−0.8 (5)	C84—C79—C80—C81	−2.3 (5)
C15—C16—C17—C18	177.9 (3)	C78—C79—C80—C81	175.9 (3)
C16—C17—C18—C19	0.9 (6)	C79—C80—C81—C82	1.4 (6)
C17—C18—C19—C20	−0.5 (6)	C80—C81—C82—C83	0.3 (6)
C18—C19—C20—C21	0.1 (6)	C81—C82—C83—C84	−1.2 (7)
C19—C20—C21—C16	0.0 (6)	C80—C79—C84—C83	1.4 (6)
C17—C16—C21—C20	0.4 (5)	C78—C79—C84—C83	−176.7 (4)
C15—C16—C21—C20	−178.4 (3)	C82—C83—C84—C79	0.3 (6)
C27—C22—C23—C24	−0.4 (6)	C90—C85—C86—C87	−0.5 (5)
C28—C22—C23—C24	178.2 (4)	C91—C85—C86—C87	179.3 (3)
C22—C23—C24—C25	0.0 (7)	C85—C86—C87—C88	0.4 (6)
C23—C24—C25—C26	0.4 (7)	C86—C87—C88—C89	−0.2 (6)
C24—C25—C26—C27	−0.3 (6)	C87—C88—C89—C90	0.1 (6)
C23—C22—C27—C26	0.5 (5)	C88—C89—C90—C85	−0.2 (5)
C28—C22—C27—C26	−178.1 (3)	C86—C85—C90—C89	0.4 (5)
C25—C26—C27—C22	−0.1 (6)	C91—C85—C90—C89	−179.4 (3)
C23—C22—C28—C33	162.9 (3)	C90—C85—C91—C92	−155.1 (3)
C27—C22—C28—C33	−18.6 (5)	C86—C85—C91—C92	25.1 (5)
C23—C22—C28—C29	−18.1 (5)	C90—C85—C91—C96	25.3 (5)
C27—C22—C28—C29	160.4 (3)	C86—C85—C91—C96	−154.5 (3)
C33—C28—C29—C30	0.2 (5)	C96—C91—C92—C93	−0.2 (5)
C22—C28—C29—C30	−178.9 (3)	C85—C91—C92—C93	−179.8 (3)
C28—C29—C30—C31	−0.8 (5)	C91—C92—C93—C94	−1.3 (5)
C29—C30—C31—C32	1.3 (5)	C92—C93—C94—C95	2.2 (5)
C29—C30—C31—C34	−177.7 (3)	C92—C93—C94—C97	−177.7 (3)
C30—C31—C32—C33	−1.3 (5)	C93—C94—C95—C96	−1.6 (5)
C34—C31—C32—C33	177.7 (3)	C97—C94—C95—C96	178.2 (3)
C31—C32—C33—C28	0.8 (5)	C94—C95—C96—C91	0.2 (5)
C29—C28—C33—C32	−0.2 (5)	C92—C91—C96—C95	0.7 (5)
C22—C28—C33—C32	178.8 (3)	C85—C91—C96—C95	−179.7 (3)
C32—C31—C34—O3	4.6 (4)	C93—C94—C97—O9	−12.0 (4)
C30—C31—C34—O3	−176.5 (3)	C95—C94—C97—O9	168.1 (3)
C32—C31—C34—C35	−175.0 (3)	C93—C94—C97—C98	166.3 (3)
C30—C31—C34—C35	3.9 (5)	C95—C94—C97—C98	−13.6 (5)
O3—C34—C35—C36	−0.6 (5)	O9—C97—C98—C99	3.5 (5)
C31—C34—C35—C36	179.0 (3)	C94—C97—C98—C99	−174.7 (3)
C34—C35—C36—O4	0.1 (5)	C97—C98—C99—O10	−3.3 (5)
C34—C35—C36—C37	−179.7 (3)	C97—C98—C99—C100	175.3 (3)
O4—C36—C37—C38	−177.6 (3)	O10—C99—C100—C101	−156.2 (3)
C35—C36—C37—C38	2.3 (5)	C98—C99—C100—C101	25.1 (5)
O4—C36—C37—C42	2.5 (4)	O10—C99—C100—C105	25.3 (5)
C35—C36—C37—C42	−177.7 (3)	C98—C99—C100—C105	−153.3 (3)
C42—C37—C38—C39	−0.6 (5)	C105—C100—C101—C102	0.0 (5)
C36—C37—C38—C39	179.4 (3)	C99—C100—C101—C102	−178.4 (3)
C37—C38—C39—C40	1.1 (6)	C100—C101—C102—C103	0.7 (6)
C38—C39—C40—C41	−0.6 (6)	C101—C102—C103—C104	−0.7 (7)
C39—C40—C41—C42	−0.3 (6)	C102—C103—C104—C105	0.0 (7)
C40—C41—C42—C37	0.8 (5)	C103—C104—C105—C100	0.7 (7)
C38—C37—C42—C41	−0.4 (4)	C101—C100—C105—C104	−0.7 (5)
C36—C37—C42—C41	179.6 (3)	C99—C100—C105—C104	177.8 (3)
C48—C43—C44—C45	0.1	C111—C106—C107—C108	−0.6 (5)
C49—C43—C44—C45	177.0	C112—C106—C107—C108	176.2 (3)
C43—C44—C45—C46	0.4	C106—C107—C108—C109	−0.1 (6)
C44—C45—C46—C47	−0.5	C107—C108—C109—C110	0.2 (6)
C45—C46—C47—C48	0.0 (5)	C108—C109—C110—C111	0.4 (6)
C46—C47—C48—C43	0.5	C109—C110—C111—C106	−1.1 (6)
C44—C43—C48—C47	−0.6	C107—C106—C111—C110	1.1 (5)
C49—C43—C48—C47	−177.5	C112—C106—C111—C110	−175.7 (3)
C48—C43—C49—C54	30.0	C107—C106—C112—C113	−26.1 (4)
C44—C43—C49—C54	−146.8	C111—C106—C112—C113	150.6 (3)
C48—C43—C49—C50	−153.1	C107—C106—C112—C117	156.3 (3)
C44—C43—C49—C50	30.1	C111—C106—C112—C117	−27.0 (5)
C54—C49—C50—C51	2.3 (5)	C117—C112—C113—C114	2.6 (5)
C43—C49—C50—C51	−174.7	C106—C112—C113—C114	−175.2 (3)
C49—C50—C51—C52	−1.2 (5)	C112—C113—C114—C115	−0.7 (5)
C50—C51—C52—C53	−0.9 (5)	C113—C114—C115—C116	−1.0 (5)
C50—C51—C52—C55	176.8 (3)	C113—C114—C115—C118	176.9 (3)
C51—C52—C53—C54	1.9 (5)	C114—C115—C116—C117	0.8 (5)
C55—C52—C53—C54	−175.7 (3)	C118—C115—C116—C117	−177.2 (3)
C52—C53—C54—C49	−0.8 (5)	C115—C116—C117—C112	1.2 (5)
C50—C49—C54—C53	−1.3 (5)	C113—C112—C117—C116	−2.8 (5)
C43—C49—C54—C53	175.7	C106—C112—C117—C116	174.9 (3)
C51—C52—C55—O5	−11.5 (5)	C116—C115—C118—O11	15.4 (5)
C53—C52—C55—O5	166.1 (3)	C114—C115—C118—O11	−162.5 (3)
C51—C52—C55—C56	169.5 (3)	C116—C115—C118—C119	−165.7 (3)
C53—C52—C55—C56	−13.0 (5)	C114—C115—C118—C119	16.4 (5)
O5—C55—C56—C57	−3.6 (5)	O11—C118—C119—C120	2.8 (5)
C52—C55—C56—C57	175.4 (3)	C115—C118—C119—C120	−176.1 (3)
C55—C56—C57—O6	1.5 (5)	C118—C119—C120—O12	0.0 (5)
C55—C56—C57—C58	−177.7 (3)	C118—C119—C120—C121	179.1 (3)
O6—C57—C58—C63	−177.9 (3)	O12—C120—C121—C122	173.0 (3)
C56—C57—C58—C63	1.3 (5)	C119—C120—C121—C122	−6.2 (5)
O6—C57—C58—C59	3.5 (4)	O12—C120—C121—C126	−8.2 (5)
C56—C57—C58—C59	−177.2 (3)	C119—C120—C121—C126	172.7 (3)
C63—C58—C59—C60	0.5 (5)	C126—C121—C122—C123	0.0 (6)
C57—C58—C59—C60	179.1 (3)	C120—C121—C122—C123	178.9 (4)
C58—C59—C60—C61	0.3 (6)	C121—C122—C123—C124	−0.2 (7)
C59—C60—C61—C62	−0.9 (6)	C122—C123—C124—C125	0.1 (7)
C60—C61—C62—C63	0.6 (7)	C123—C124—C125—C126	0.2 (7)
C61—C62—C63—C58	0.2 (6)	C124—C125—C126—C121	−0.5 (6)
C59—C58—C63—C62	−0.8 (5)	C122—C121—C126—C125	0.4 (6)
C57—C58—C63—C62	−179.3 (3)	C120—C121—C126—C125	−178.5 (4)

### Hydrogen-bond geometry (Å, °)

**Table:**

*D*—H···*A*	*D*—H	H···*A*	*D*···*A*	*D*—H···*A*
O1—H1A···O2	1.20 (5)	1.33 (5)	2.480 (4)	156 (4)
O3—H3A···O4	1.21 (4)	1.33 (4)	2.479 (4)	156 (3)
O5—H5A···O6	1.33 (5)	1.22 (5)	2.474 (4)	152 (4)
O7—H7A···O8	1.28 (5)	1.26 (5)	2.465 (4)	152 (4)
O9—H9A···O10	1.20 (5)	1.34 (5)	2.473 (4)	154 (3)
O11—H11A···O12	1.33 (5)	1.25 (5)	2.494 (4)	152 (4)
C122—H122···O10^i^	0.93	2.58	3.429 (5)	152
C19—H19···Cg10^ii^	0.93	2.93	3.739 (5)	147
C23—H23···Cg17^iii^	0.93	2.90	3.714 (4)	146
C32—H32···Cg17^iv^	0.93	2.94	3.749 (4)	147
C39—H39···Cg3^i^	0.93	2.82	3.674 (5)	152
C48—H48···Cg11^v^	0.93	2.79	3.618 (4)	149
C69—H69···Cg8^i^	0.93	2.95	3.820 (4)	155
C93—H93···Cg2^vi^	0.93	3.00	3.692 (3)	133
C107—H107···Cg14^i^	0.93	2.83	3.670 (4)	151

Symmetry codes: (i) *x*−1, *y*, *z*; (ii) *x*+1, *y*, *z*; (iii) −*x*, −*y*, −*z*+1; (iv) −*x*+1, −*y*, −*z*+1; (v) −*x*+1, −*y*+1, −*z*+1; (vi) −*x*+2, −*y*, −*z*+1.

## References

[bb1] Bertolasi, V., Cilli, P., Ferretti, V. & Gilli, G. (1991). *J. Am. Chem. Soc.* **113**, 4917–4925.

[bb2] Bruker (1997). *SMART* Bruker AXS Inc., Madison, Wisconsin, USA.

[bb3] Bruker (1999). *SAINT* Bruker AXS Inc., Madison, Wisconsin, USA.

[bb4] Desiraju, G. R. & Steiner, T. (2001). *The Weak Hydrogen Bond In Structural Chemistry and Biology. *IUCr Monographs on Crystallography No. 9. Oxford University Press.

[bb5] Gilli, P., Bertolasi, V., Pretto, L., Ferretti, V. & Gilli, G. (2004). *J. Am. Chem. Soc.* **126**, 3845–3855. 10.1021/ja030213z 15038739

[bb6] Hasegawa, E., Ishiyama, K., Fujita, T., Kato, T. & Abe, T. (1997). *J. Org. Chem.* **62**, 2396–2400. 10.1021/jo9622439 11671572

[bb7] Jang, H., Shin, C. H., Jung, B. J., Kim, D. H., Shim, H. K. & Do, Y. (2006). *Eur. J. Inorg. Chem.* **4**, 718–725.

[bb8] Sheldrick, G. M. (1996). *SADABS* University of Göttingen, Germany.

[bb9] Sheldrick, G. M. (2008). *Acta Cryst.* A**64**, 112–122. 10.1107/S0108767307043930 18156677

[bb10] Spek, A. L. (2003). *J. Appl. Cryst.* **36**, 7–13.

[bb11] Vila, A. J., Lagier, C. M. & Olivieri, A. C. (1991). *J. Phys. Chem.* **95**, 5069–5073.

